# Comparative Analysis of Gum Resins

**Published:** 1811-04

**Authors:** Henry Braconnot

**Affiliations:** Professor of Natural History, &c.


### *Comparative Analysis of Gum Resins: by Mr.* Henry Braconnot, *Professor of Natural History, &c.*

[Nicholson's Jour.]

### *Analysis of Gamboge.*

*Sect.* I. If gamboge be exposed to the flame of a candle, it swells up, and burns like a resin. Heated in a capsule it emits a peculiar smell, softens, and is decomposed before it melts.

Fifty gram. [772 grs.] exposed to distillation produced,

1st, a brown water, containing empyreumatic acetic acid:

2d, a small quantity of a light oil:

3d, afterward came over in considerable quantity another oil, heavy, thick, and brown.

In the retort remained a light coal, weighing 8 gram. [123·5 grs]. It was incinerated with difficulty, and left 5 decig. [7·7 grs] of ashes, which yielded 2 cent. [0·3 of a gr.] of potash in part sulphated, 4 cent. [0·6 of a gr.] of phosphate phate of lime, 6 cent. [0·9 of a gr.] of carbonate of lime, and 3 dec. [4·6 grs] of quartz sand, containing a little charcoal, and some traces of oxide of iron. No ammonia was found in the liquid products.

*Sect.* II. Twenty gram. [309 grs] were treated with hot alcohol and filtered. What remained on the filter, after being well washed with alcohol, was a grayish substance, that dried with difficulty, and was then brittle. In this state it weighed exactly 4 gram. [61·7 grs]; had a faint taste, or was nearly insipid; and dissolved entirely in water, except a decig. [1·5 gr.] of impurities. The solution reddened litmus. Evaporated to dryness it left a transparent, friable residuum, resembling the high coloured gum of the plum-tree, burning like it with little flame, and leaving a considerable quantity of a compact coal, in which was some phosphate of lime.

The alcoholic solution was red. Evaporated to dryness it afforded 16 gram. [247 grs] of resin. This was transparent, red, without any perceptible taste, and pretty decidedly electric by friction. When powdered it emitted a peculiar smell, and assumed a bright yellow colour.

On pouring water into a saturated solution of this resin in alcohol, there is a sensible evolution of heat, and a uniform, yellowish, milky liquid is produced; while most other resins are precipitated from alcohol in clots.

*Sect.* III. Solution of potash acts very quickly on the resin of gamboge, particularly if heated. The result is a red liquid of an oily appearance, in which the properties of the potash are neutralized. On evaporating this compound almost to dryness, it crystallizes like the solution of aloes.

The soap or saponule of this resin is of a deep red approaching to black, and feels greasy between the fingers. When dried it is friable, and resembles a resin. It has the taste of rancid fat, leaving a slight sensation of acrimony at the root of the tongue. It is easily soluble in water, without rendering it turbid. Acids precipitate this solution in such abundance, that the whole becomes a thick coagulum of a fine yellow colour. Lime-water throws down from it a fine orange colour precipitate. Earthy salts and most solutions of the white metals likewise produce yellow precipitates in it. It precipitates sulphate of iron brown, and nitrate of copper green.

*Sect.* IV. Ten gram. [154·4 grs] of the resin of gamboge were put into a retort with 80 parts of strong nitric acid of the shops. As soon as the retort felt the heat of the fire, red vapours arose, the intensity of which soon disappeared. The first product was returned into the retort, and the operation continued, till the matter was dissolved, and the solution reduced to the consistence of a sirup*. On cooling a mass of lamellar crystals was formed, enveloped in a viscid matter. The whole being diluted with a quantity of water, a sediment was deposited, which, when well washed and dried, weighed 1·3 gram. [20 grs].

This matter is of a yellowish colour, and bitter taste. It is partly soluble in boiling water. On cooling the solution grows turbid, and lets fall a portion. The filtered solution is of a reddish yellow, froths when shaken, reddens infusion of litmus, is rendered of a deeper colour by the addition of an alkali, and forms a slight precipitate after some time with sulphate of iron.

On burning coals this substance does not melt so easily as the resin, diffuses a fragrant smell, and leaves a great deal of coal.

It combines very well with potash and spirit of wine, forming with them transparent red solutions.

Nitric acid, heated gently with it, dissolves it, without occasioning any sensible alteration. Water produces a copious white coagulum in the solution.

From these properties I think I may fairly consider this substance as a particular species of soluble factitious resino-amer, combined with a yellowish resiniform substance insoluble in water.

The acid liquor and waters of elutriation were boiled down, to drive off any nitric acid that might remain; and the residuum was diluted with water, in which a small quantity of potash was dissolved, which separated 4 dec. [6 grs] of the yellow resiniform substance. The liquor being again boiled down, then treated with alcohol, and filtered, left 1 gram. [15.4 grs] of very white superoxalate of potash. The alcoholic solution produced on evaporation 3 gram. [46·3 grs] of bitter matter, soluble in water, and containing malic acid.

*Sect.* V. I diluted some of the resin of gamboge in fine powder with water; and passed into it a stream of oximuriatic acid gas, to try its effect on the colour; and in fact it destroyed its fine yellow. The milky liquor being boiled down, then diluted with water, and filtered, left on the filter a sub-

* The product of this distillation, being thoroughly saturated with chalk, and distilled anew, yielded a slightly acid liquor, of a very pungent smell, and bitter to the taste. Alkalis gave it a light yellow tinge. On adding sulphate of iron to this mixture a precipitate was formed, which was completely soluble in acids; so that there was no prussic acid in this liquid, but I am not fully acquainted with its nature.

stance, stance, which was washed with boiling water, till what came off would no longer redden litmus. The following were its properties.

It is pulverulent, of a pale yellow colour, and without any perceptible taste. It crackles between the teeth like an insoluble salt, and boiling water will not dissolve it. It is very little fusible, and emits no smell, till it begins to be decomposed; but if it be set on fire, or thrown on burning coals, it emits pungent fumes of muriatic acid.

Weak acids separate nothing perceptible from it; but with concentrated acids charcoal and muriatic acid are produced.

Combined with potash the compound has a pleasant soapy smell, and nitrate of silver throws down from its solution a precipitate partly soluble in nitric acid.

I distilled 6 gram. [92·6 grs] of this substance in a small retort, which was heated to redness. The product was collected in a few decigr. [about $\frac{1}{7}$ of an oz. measure each] of water, which, being examined toward the end of the distillation, was very sour, and had the smell of muriatic acid. To this water I added nitrate of silver, which produced a copious curdy precipitate of muriate of silver. This precipitate weighed 5·4 gr. [83·4 grs], which would contain 1·35 gram. [20·84 grs] of muriatic acid, according to the proportions given by Bergman of 25 acid to 75 oxide.*

In the retort were left 2·1 gram. [32·4 grs] of a tumid coal.

Hence it follows, that 100 parts of this acidiferous resinous substance were composed of

| | |
|---|---|
| Dry muriatic acid . . | 22·5 |
| Charcoal . . . . . | 35 |
| Oxigen, hidrogen, and carbon | 42·5 |
| | 100 |

*Sect.* VI. Thus it appears, that gamboge is a true gum-resin, since we find in it a peculiar resin very well marked, and a gum resembling that of many of our fruit trees.

* This certainly estimates the acid too high. From 19 to 19·5 per cent of acid is the most that can be allowed, according to the experiments of several of the most eminent modern chemists.

### *Analysis of Euphorbium.*

*Sect.* 1. The Dutch have their euphorbium from Malabar, where it flows from the euphorbia antiquorum; that used in England is from the euphorbia canariensis,* which furnishes the euphorbium used in France, as Mr. Braconnot had an opportunity of satisfying himself, by finding several branches of the tree among the specimens he examined.

*Sect.* II. Euphorbium, when exposed to a gentle heat, softens easily, and loses a twentieth of its weight in moisture. I boiled 4 gram. [61·8 grs] in 100 gram. [1544 grs] of distilled water. The filtered liquor left an insoluble substance, which, when dried, weighed 3 gram. [46·3 grs]. What passed through was of an amber colour, and had a bitter taste with a slight degree of acrimony.

This solution reddened infusion of litmus. Oxalate of potash threw down from it a pretty copious precipitate of oxalate of lime. Nitrate of lead formed in it a white precipitate entirely soluble in distilled vinegar. Lime-water rendered it turbid, and occasioned a yellow precipitate, which vinegar dissolved.

*Sect.* III. *A*. I boiled 20 gram. [308·8 grs] of euphorbium in 90 gram. [1389·6 grs] of alcohol at 36° [spec. grav. 0·837], which was sufficient to dissolve all the parts capable of solution. This solution filtered at a boiling heat left a substance on the filter, which, after being well washed with alcohol and dried, weighed 6·4 gram. [98·8 grs].

*B*. Having mixed together the alcoholic solutions, which had grown turbid on cooling, and let them stand at rest for two days, a considerable quantity of white, granular, and somewhat gelatinous substance was deposited, which, being washed with alcohol and dried, weighed 4·7 gram. [72·5 grs]. It still retained some alcohol, which being driven off by heat, only 3·4 gram. [52·5 grs] remained. This substance was semitransparent, capable of being indented by a hard body, softened readily between the fingers, was almost wholly volitalized on a red-hot iron, and comported itself like bees-wax, which it resembles in smell when melted or burned. This wax of euphorium retained a slight degree of acrimony, no doubt because it had not been sufficiently washed with alcohol. I made a taper of it, which burned with a very clear flame.

* According to our college, who have readmitted it into their last Pharmacopœia, from the euphorbia officinarum.

*C*. The

*C.* The 6·4 gram. [98·8 grs] insoluble in alcohol (*A*) were heated to the boiling point in 100 gram. [1544 grs] of distilled water. The filtered liquor left behind little bits of wood and thorns, which when dried weighed 2·7 dec. [4·17 grs].

*D.* The aqueous solution (*C*), being evaporated, formed a varnish on the surface of the glass. On evaporating to dryness a brittle substance was obtained, which separated in micaceous scales, did not attract moisture, and weighed 4·1 gram. [63·3 grs], which I perceived at once to be malate of lime.* In fact, on heating this substance with diluted sulphuric acid, I obtained, 1st, very white sulphate of lime, which, after being washed and dried, weighed 1·6 gram. [24·7 grs] : 2dly, an acid, which alcohol dissolved, and from which it separated 5 dec. [7 grs] of sulphate of lime. The solution, being evaporated, produced 2 gram. [30·83 grs] of malic acid, retaining a little sulphuric acid, which was separated by barytes.

Malate of lime therefore appears to exist in tolerable quantity in the milky juice of the euphorbias; and it was this salt, which the older chemists mistook for a gum in the euphorbium of the shops, and which Mr. Laudet has confounded with extract.

*E.* The alcoholic solution (*B*), being evaporated to dryness, left a residuum weighing 8·3 gram. [128 grs]. This was treated with cold alcohol, which dissolved the resinous parts, and farther separated 4 dec. [6.2 grs] of wax.

*F.* This solution being evaporated afresh, a resin was obtained, that attracted moisture from the air in a small degree. This was owing to the presence of malate of potash, which I obtained by heating the resin with distilled water. When well dried it weighed 4 dec. [6·2 grs].

*G.* The resin of euphorbium has a reddish transparency, and extreme acrimony, whence it may be considered as a violent poison. It becomes electric by friction. Alkalis have no sensible action on it. Sulphuric acid dissolves it cold. Treated with nitric acid at a heat of 20° [77° F.] it softens, grows yellow, and begins to decompose. By increasing the heat, a complete solution is obtained, which, when evaporated, yielded a great deal of yellowish resiniform matter, a soluble resinoamer substance, and some traces of oxalic acid.

---

* I have already made known, that the ricinus, which is of the family of euphorbias, contains malic acid, neutralizing a large quantity of potash and lime.

Sect.

*Sect.* IV. From these experiments it follows, that 100 parts of euphorbium are composed of

| | |
|---|---|
| Water . . . | 5 |
| Wax . . . | 19 |
| Woody matter . | 13·5 |
| Malate of lime . . | 20.5 |
| Resin . . . | 37 |
| Loss . . | 3 |
| | 100 |

